# A125 DOES AI INFLUENCE ADENOMA DETECTION RATES IN FIT-POSITIVE PATIENTS

**DOI:** 10.1093/jcag/gwae059.125

**Published:** 2025-02-10

**Authors:** B S McGrath, M Borgaonkar, G S McGrath

**Affiliations:** Memorial University of Newfoundland, StJohn’s,, Canada; Memorial University of Newfoundland, StJohn’s,, Canada; Memorial University of Newfoundland, StJohn’s,, Canada

## Abstract

**Background:**

The Fecal Immunochemical Test (FIT) is a screening tool that identifies patients more likely to harbor adenomas or colorectal cancer (CRC). Rates of adenoma detection during colonoscopy can vary significantly among endoscopists. Artificial Intelligence (AI) has been shown to improve adenoma detection during colonoscopy.

**Aims:**

To assess if AI assistance during colonoscopy can improve adenoma detection in FIT-positive patients.

**Methods:**

In October and November of 2023, AI was utilized during colonoscopy for FIT-positive patients at Eastern Health, Newfoundland. The 61 FIT-positive patients who had colonoscopy with AI assistance were compared to 61 FIT-positive age, gender, and endoscopist-matched controls who had colonoscopies performed without AI assistance during the preceding 6 months. Demographic data were collected on all patients as well as colonoscopy findings, including CRC detection, polyp detection, and histology. The primary outcome was the proportion of patients with adenomas in the two groups. Secondary outcomes included advanced adenoma detection rate and sessile serrated lesion detection rate. Sample size was one of convenience as only 61 FIT-positive patients had colonoscopies with AI assistance while AI was temporarily available at our institution. Data were entered in SPSS version 17 for analysis. A chi-squared test was used to compare proportions. The study received approval from the local Health Research Ethics Board.

**Results:**

122 colonoscopies performed by 13 endoscopists (7 General Surgeons, 6 Gastroenterologists) were included. Sixty-eight patients (55.7%) were female and fifty-four patients (44.3%) were male with an average age of 64 (SD = 6.729). Of the patients who underwent AI-assisted colonoscopies, 73.8% had adenomas. Of the patients who underwent colonoscopy without AI assistance, 63.9% had adenomas. There was a non-significant trend toward higher adenoma detection in the patients who had a colonoscopy with AI (x^2=5.165, p=0.076). AI-assisted colonoscopies found advanced adenomas in 44.3% of patients compared to 29.5% in the no AI group (x^2=3,020, p=0.221). In the AI group, 8.2% of patients had sessile serrated lesions (SSL), compared to 1.6% in the standard procedure group (x^2=2.805, p=0.094).

**Conclusions:**

This study showed a trend toward higher adenoma, advanced adenoma, and SSL detection with AI assistance in FIT-positive patients. Repeating this study with a larger sample size might clarify the effect of AI assistance during colonoscopy in FIT-positive patients

**Funding Agencies:**

None

